# Determining the effects of traditional learning approach and interactive learning activities on personal and professional factors among Saudi intern nurses

**DOI:** 10.1002/nop2.633

**Published:** 2020-09-30

**Authors:** Ahmad A. AlKhaibary, Faten Z. Ramadan, Ahmad E. Aboshaiqah, Omar G. Baker, Salwa Z. AlZaatari, Salim Z. AlZaatari

**Affiliations:** ^1^ King Saud Medical City Riyadh Saudi Arabia; ^2^ King Saud University Riyadh Saudi Arabia; ^3^ Lebanese American University Beirut Lebanon; ^4^ American University for Science and Technology Beirut Lebanon

**Keywords:** interactive learning activity, intern nurses, personal factors, professional factors, traditional learning activity

## Abstract

**Aim:**

This study determines the impact of traditional and interactive learning activities on personal and professional development among Saudi intern nurses.

**Design:**

A comparative research design was adopted by recruiting 48 intern nurses, who were divided into two equal groups.

**Methods:**

Data were collected through the clinical assessment tool for nursing education.

**Results:**

Enthusiasm (*p* < .0001), initiative (*p* < .0001), realistic self‐confidence (*p* < .0001), competent cooperation with staff (*p* < .0001) and competent cooperation with patients (*p* < .0001) were significantly higher among the interactive learning activity group compared with traditional group. The interactive learning activity showed better attitude of Saudi intern nurses in terms of caring, respect and sensitivity towards the needs and well‐being of their patients, while considering personal factors.

## 1 INTRODUCTION

The role of nursing educators has become increasingly important when it comes to student's learning and establishing their problem‐solving abilities (Yew & Goh, 2016). Internet is among the fastest growing and penetrated technologies across the world. Nursing education progresses the use of technology, including e‐learning in both the theoretical and clinical simulation courses (Betihavas et al., 2016). The intern nurses were subjected to different kinds of stressors, which are often observed in academic, clinical, social and interpersonal areas (Alsaqri, 2017). Students entering university from secondary schools’ lack confidence required to learn and adapt to the challenges (Hassel & Ridout, 2018).

Interactive learning uses two or more complimentary approaches for teaching the same material, based on web‐based modules, activities, classroom lectures and discussions (Hwang et al., 2015). The obligation of nurse educators creates learning environments that encourage critical thinking. The transformation of traditional teaching methods should be based on the improvement of learning experiences and facilitate lifelong learning (Sharma, 2017). Teaching activities will promote further significant learning that involves experience through execution and communication with others. Creativity can be established as innovations that were beneficial for students and the teachers, where originality was associated to achieve some type of tangible end (Sinay et al., 2017).

The use of interactive methods in nursing management enhances education, strengthens governance, galvanizes the effort and empowers individuals for achieving the human development goal (Mendes et al., 2016). The interactive learning strategy of teachers offers a new modality and methodology for learning and teaching as it reduces the amount of direct instruction and maximizes one‐to‐one interaction in the teaching process (Hampton et al., 2017). It can also enhance and support teamwork, cultural diversity and social interaction among students. The responsibilities of students have a subsequent change from passive to active participants. The interactive learning is increasingly implemented in the broader educational sphere, improving student's ownership of learning by being more interactive throughout actual class time (Alshammari et al., 2018). Determinants of interactive learning recommend that this pedagogy offers students to learn at their own pace and provides flexibility to involve with the electronic resources. There should be vigorous and sustainable implementation of E‐learning needs into the local educational context based on the national strategies and other national interventions and efforts (Barteit et al., 2019).

Negative clinical experiences have more effects on attitude, students learning and trust in clinical setting compared with positive clinical experiences. A great deal of time and energy can be wasted by negative clinical experiences and factors contributing to the learning of these experiences such as lack of role clarity, lack of knowledge among teachers and learners and workload (Baraz et al., 2015; Flott & Linden, 2016). However, the implementation of interactive activities can be successful through different instructional activities such as include role playing and simulation, small group discussion, brainstorming and jigsaw methods. Brainstorming indicates the procedure where students develop a list of problems for a question or topic, whereas evaluation of the responses was initially deferred (Hidayanti et al., 2018). Role playing is an effective technique to engage students and allow them to involve with their peers as they attempt to complete the task allocated to them (Dyson et al., 2016). This task can be completed in cooperative groups, and students can retain their individual role in the class period. However, the involvement of students is much more important as they make efforts for reacting to the material from the insights of their character (Darling‐Hammond et al., 2020). Teachers can teach the same material by combining discussions, classroom lectures, activities and/or web‐based modules (Hsu & Hsieh, 2011; Mersel & Mersel, 2014).

Formation of an interactive learning environment supports both the learners and preceptors to verbalize their objectives and requirements in learning and accomplish a mutual interest to improve the clinical competencies of the learners (García‐Carrión et al., 2018). This assists students to identify their potential to be proficient nurses in the future. The educators can achieve a new teaching method that will be more proficient for intern nurses to become independent nurses. It is believed that interactive learning activity can improve the formal and informal nursing education (Dunn & Milheim, 2017). It is also substantial to nursing faculty, nursing managers and nursing students as it aims to continue the transition process of intern nurses into staff nurse role to deliver excellent care to patients and their families and improve patient safety with high quality. The interactive learning activity method intends to develop competent nurses using motivation and empowerment (Hassankhani et al., 2014). The establishment of interactive learning activity is encouraged from the challenges of traditional learning method in clinical environment. The use of rubrics can expand interactive learning activity in validating the clinical assessment. Clinical assessment remains a challenge, even for temporary faculty. The objectives of clinical courses are differently interpreted by students and faculty (Isaacson & Stacy, 2009). Flipped learning is one of the approaches for technological integration into the classroom. This pedagogical approach has become progressively renowned, and there is an elevating body of literature to examine the integration of this teaching methodology in different classrooms (Eppard & Rochdi, 2017). The flipped classroom teaching model is explained as one where the activities conventionally performed by students outside class are switched into the classroom session, while what is conventionally done in class (Tan et al., 2017).

A professional learning community is explained as a bunch of principals, researchers, teachers and administrative staff, working collaboratively for progressively enhancing and developing students’ learning (Ellerani & Gentile, 2013). The core features of professional learning community can be explained by the decision‐making and collaboration among peers and their democratic behaviour (Jagtap, 2015). Other attributes of professional learning communities are defined through the strategies activated by different elements in the community such as shared vision, beliefs and values; peer support; sustainable development; positive climate conditions; trust; cooperative learning among adults; distributed and supportive leadership; and continued self‐assessment (Ellerani & Gentile, 2013). Several scholars have commenced to stress greater focus on collaborative teacher learning and job‐embedded learning as research explicates the understanding on teachers’ learning (Fessehatsion, 2017).

In this context, the study aims to determine the impact of traditional and interactive learning activities on personal and professional development of Saudi intern nurses. Interactive learning activity is introduced in this study as a clinical learning approach for Saudi nursing interns throughout their internship training. The findings of this study can serve as a guiding framework to design and plan interactive learning practices to solid educational foundations. Following research questions were drawn to fulfil the aim of this study:

- **Question 1:** What is the impact of traditional and interactive learning activities on personal and professional development of Saudi intern nurses?
- **Question 2:** To what extent interactive learning activity can be effective for Saudi nursing interns throughout their nursing training?

## 2 MATERIAL AND METHODS

### 2.1 Research design

A comparative research design was adopted to measure the effects of traditional learning and interactive learning. Classroom‐based learning, lecturing and preceptorship were included as traditional learning activities, whereas e‐learning, flipped classroom, group learning activities, simulation and preceptorship were included as interactive learning activities. The content assessment protocol was established similarly to the one implemented for evaluating the contents in the classroom. The typical established protocol was for teaching a subject and then assessing the extent of content learning in the classroom of fifth year of the nursing education in the country of the authors in this work.

### 2.2 Setting

Intern nurses were selected from two tertiary hospitals in Riyadh, Saudi Arabia. The nurses from first hospital were provided with interactive learning activities, whereas nurses from other hospital were provided with traditional learning activities.

### 2.3 Population and Sampling

The study population included Saudi intern nurses enrolled in the fifth year of their degree in nursing. The inclusion criteria were nurses having Saudi nationality, identified as interns by the affiliated schools and healthcare institutions and joined the National Internship Program for 12 months. The nurses were randomly selected from different clinical areas such as paediatric, critical care, medical surgical and maternity.

The estimated sample size for this study must be 48 or more based on 95% confidence interval (CI) and medium effect size of 0.25 for a significance level of α = 0.05. Therefore, this study has randomly selected 60 nurses, who were equally divided into two groups. However, after exclusion of few nurses due to their unwillingness, the final sample comprised of 48 nurses with 24 nurses in each group. This ratio accounted for response rate of 80%.

### 2.4 Instrument

The Clinical Assessment Tool for Nursing Education (CAT‐NE) proposed by Skúladottir and Svavarsdottir (2016) was used as study instrument. This assessment tool was appropriate for evaluating the clinical performance of nursing students as it comprises of rubrics that list the criteria for the expected performance of students. Learning objectives were clarified using this assessment tool along with improving the emphasis of the assessment process and making evaluation more objective‐oriented.

The assessment comprises of 13 items, which were categorized as either personal or professional factors. There were 6 items for personal factors: enthusiasm, initiative, realistic confidence, competence with staff, competence with clients and caring. Professional factors include 7 items: documentation; informing and educating patients; clinical competence; patient assessment and care; theoretical knowledge; critical thinking; and self‐control.

### 2.5 Data analysis

The Statistical Package for Social Sciences (SPSS) version 21 was used to analyse the data. The scores of the participants in personal and professional development were determined using descriptive statistics. T test was used to determine the difference between traditional and interactive learning methods between both the groups.

## 3 RESULTS

A total of 48 participants were enrolled in this study, which were divided into two groups. Table 1 shows the information related to profile characteristics of the participants. Most participants in both groups were female (66.7%, Group 1) and (83.3%, Group 2). Most participants in Group 2 were 23 years old (66.7%), while 50% of the participants were 22 years old. Participants in both the groups completed their 1‐year internship.

**Table 1:** Profile characteristics of the participants (*n* = 48)

Item	Characteristics	Group 1‐ TLA *n* (%)	Group 2‐ ILA *n* (%)
Age	22	12 (50)	6 (25)
	23	10 (41.7)	16 (66.7)
	24	2 (8.3)	2 (8.3)
Gender	Male	8 (33.3)	4 (16.7)
	Female	16 (66.7)	20 (83.3)
School	A	14 (58.3)	6 (25)
	B	9 (37.5)	10 (41.7)
	C	0 (0)	1 (4.2)
	D	0 (0)	1 (4.2)
	E	0 (0)	1 (4.2)
	F	1 (4.2)	1 (4.2)
	G	1 (4.2)	1 (4.2)
	H	1 (4.2)	0 (0)
	I	0 (0)	1 (4.2)
Duration	12 months	24 (100.0)	24 (100.0)
	<12 months	0 (0)	0 (0)
Marital status	Single	24 (100.0)	23 (95.8)
	Married	0 (0)	1 (4.2)

Table 2 presents comparison of the CAT‐NE scores between traditional and interactive learning activity for personal factors using independent sample *t* test. The mean score for caring was significantly higher among interactive learning activity group (mean = 4.5, *SD* 0.7) compared with traditional group (mean = 2.7, *SD* 0.9). The findings have shown a significant difference between both groups, in terms of caring factor (t (46) = 8.30, *p* < .0001). Furthermore, enthusiasm (t (46) = 8.14, *p* < .0001), initiative (t (46) = 7.16, *p* < .0001), realistic self‐confidence (t (46) = 8.03, *p* < .0001), competent cooperation with staff (t (46) = 7.39, *p* < .0001) and competent cooperation with patients (t (46) = 7.71, *p* < .0001) were significantly higher among interactive learning activity group, compared with traditional group.

**Table 2:** Comparison of the CAT‐NE Scores (Personal Factors)

Variable	Group 2‐ TLA	Group 1‐ ILA	*t*	*p*
	Mean, *SD*	Mean, *SD*		
Caring	2.7 (0.9)	4.5 (0.7)	8.30	<.001
Enthusiasm	2.2 (1.1)	4.5 (0.8)	8.14	<.001
Initiative	2.3 (1.3)	4.5 (0.7)	7.16	<.001
Realistic self‐confidence	2.2 (1.1)	4.5 (0.7)	8.03	<.001
Competent cooperation with staff	2.2 (1.2)	4.5 (0.8)	7.39	<.001
Competent cooperation with patients	2.2 (1.1)	4.5 (0.8)	7.71	<.001

Table 3 presents comparison of the CAT‐NE scores between traditional and interactive learning activity for professional factors using independent sample *t* test. The mean score for theoretical knowledge was significantly higher among interactive learning activity group (mean = 3.8, *SD* 0.6) compared with traditional group (mean = 1.6, *SD* 1.2). The findings have shown a significant difference between both groups, in terms of theoretical knowledge factor (t (46) = 7.13, *p* < .0001). Furthermore, critical thinking (t (46 = 8.14, *p* < .0001), self‐control (t (46) = 6.21, *p* < .0001), patient assessment and patient care (t (46) = 8.36, *p* < .0001), clinical competence (t (46) = 8.42, *p* < .0001) and informing and educating patient with patients (t(46) = 8.34, *p* < .0001) were significantly higher among interactive learning activity group, compared with traditional group.

**Table 3:** Comparison of the CATNE Scores (Professional Factors)

Variable	Group 2‐ TLA	Group 1‐ ILA	*t*	*p*
	Mean (*SD*)	Mean (*SD*)		
Theoretical knowledge	1.6 (1.2)	3.8 (0.6)	7.13	<.0001
Critical Thinking	1.7 (1.2)	3.8 (0.8)	6.89	<.0001
Self‐control	1.6 (1.1)	3.7 (0.9)	6.21	<.0001
Patient assessment/patient care	1.7 (1.1)	4.1 (0.7)	8.36	<.0001
Clinical competence	1.6 (1.2)	4.0 (0.7)	7.98	<.0001
Documentation	1.7 (1.1)	4.1 (0.6)	8.42	<.0001
Informing/Educating Patients	1.5 (1.2)	4.1 (0.7)	8.34	<.0001

## 4 DISCUSSION

The present study showed that interactive learning activity had positive impact on the intern nurses, compared with traditional learning activity. The interactive learning activity showed better attitude of respondents in terms of care, respect and sensitivity towards the needs and well‐being of their patients. It was also shown that these nurses were enthusiastic and keen for learning opportunities. The intern nurses also showed independence in working and were more confident. They were confident, more responsible, flexible, conscientious and functional even when exposed to new projects. The nurses exposed to interactive learning activity were more skilled in nursing evidence‐based practice and treatments. Critical thinking skills have been frequently used by nurses; therefore, they were more disciplined and organized. They provided appropriate education and showed excellence in nursing documentation. Interactive learning activity has assisted intern nurses to develop clinical competence of a licensed nurse.

These findings were somewhat supported by one of the previous studies stating that learners would benefit from a blended learning model, comprising of traditional‐style classroom lectures with e‐learning aspects, compared with a sudden switch to e‐learning (Mersal & Mersal, 2014). Furthermore, it is argued that intern nurses were more comfortable with traditional‐style teaching where the teacher takes control of everything in the classroom. These nurses were likely to face hard time to switch to blended learning, where they must play a more active role in the classroom (Hsu & Hsieh, 2011). Previous study has also asserted that the successful implementation of the e‐learning can be obstructed by restricted access to computing facilities (Barteit et al., 2019).

The learning experiences of nursing students in medical/surgical courses can be improved by the flipped classroom; however, there are challenges associated with this transformative process (Tan et al., 2017). Students must convert from passive learning to active learning by becoming more independent and autonomous. Use of new technology can be challenging and intimidating process for faculty and students, and much effort was required to reduce frustration (Eppard & Rochdi, 2017). These approaches were designed to make the medical‐surgical content more meaningful, engaging and encourage active learning to stimulate clinical reasoning, assist students and make associations between theory to practice. Overall, nurses did not like this transformative teaching strategy and reacted with discomfort and anxiety in teaching format. Another contributing factor to this discomfort was the lack of preparation and the lack of selection to participate in the new teaching method. Relationship building is an emotional practice, and some teachers have explained an inherent susceptibility associated with the procedure to develop trustworthy interpersonal associations with their students. In this study, teachers demonstrated that developing close associations may lead to a greater exposure to sensitive disclosures and negative emotions among students.

The nurses were not provided education about different assessment methods to evaluate their self‐directed learning, nursing activities, illness and injuries and the quality of nursing service in their wards. The present study showed that the clinical performance of intern nurses was perceived by preceptors as facilitators of clinical performance. This was the first study in Saudi Arabia to explore a new clinical teaching method and compare with the traditional approach. The study recommends that teaching strategies must be assessed periodically to derive the most adequate and effective teaching plan. It supports researchers and academicians to explore the uniqueness of clinical teaching–learning process specifically that the new generation was highly distinctive from the past learners. Integrating innovative teaching approach in clinical setting was observed as important in this study to achieve several health care‐related objectives in the upcoming Saudi 2030 vision. The mixture of e‐learning and traditional learning was suggested, although interactive learning was dominant in this study.

The main strength of this assessment tool was that it has been constructed through collaboration with those who use it such as clinical teachers, nursing students, supervisory teachers and clinical expert teachers as it improves its relationship to actual clinical behaviour and elevates its validity (Isaacson & Stacy, 2009). Another strength of this assessment tool was that the same tool can be used for evaluating clinical performance using the overall learning process, reinforcing its validity and continuity. The supervisory teachers have approved this tool and applied in most of the clinical courses in Saudi schools.

The limitation of the validation of the CAT‐NE assessment tool was that it cannot predict the limited number of students who have failed clinical study. Therefore, it is result of the integration of the assessment tool or reluctance of clinical teachers to fail students. In this regard, additional research was needed to explore this issue.

## 5 CONCLUSION

The study has compared the effects of clinical teaching strategies including traditional and interactive learning activity among Saudi intern nurses based on the 13 assessment items. The study concluded that the overall scores between the two groups were significantly different, where the intern nurses exposed to interactive learning activity scored significantly higher. The importance of professional development was observed from theoretical knowledge, critical thinking, self‐control, patient assessment and patient care, clinical competence and documentation and informing. The activities in interactive learning were admirable to develop new nurses towards becoming clinically competent and to satisfy clinical performance.

The findings of this study have shown that both professional and personal development among intern nurses were significantly confirmed with interactive learning compared with traditional clinical teaching. The nurses’ educators were suggested to explore scientifically updated teaching methods such as interactive clinical teaching, addressing the learning needs of the new breed of nurses. The success in nursing practice relies on the clinical competence achieved by the nurse. Therefore, the progression of the nursing skills, attitude and knowledge of the nurses, specifically among new graduates or interns, is important. The intern nurses and nurses are supported to use interactive learning to development of nursing personal and professional competence. The study recommends that future studies must be geared towards the progression of a highly acceptable pedagogy among Saudi nurses to enhance their commitment, performance and motivation in the clinical environment.

## CONFLICT OF INTEREST

The authors declare no conflict of interest.

## AUTHOR CONTRIBUTIONS

AAK: Correspondence, analysis, literature, data, drafting, editing and finalization. FZR, AEA, OGB, SZZ and SZA: Drafting and analysis.

## Data Availability

The data are available upon request.
